# S2-HGNN: Scale-Aware Hypergraph Node Classification with Spectral Inductive Bias

**DOI:** 10.3390/e28060592

**Published:** 2026-05-26

**Authors:** Jiangnan Zhou, Sheng Zhang, Bing Wu, Qiuming Wang, Chennan Wu, Ziqiang Luo, Ka Sun, Hongmei Mao

**Affiliations:** School of Information Engineering, Nanchang Hangkong University, Nanchang 330063, China; 2404085404312@stu.nchu.edu.cn (J.Z.); 2404085410302@stu.nchu.edu.cn (B.W.); 2404085404315@stu.nchu.edu.cn (Q.W.); 2404085404323@stu.nchu.edu.cn (C.W.); 2304085410004@stu.nchu.edu.cn (Z.L.); 70308@nchu.edu.cn (K.S.); maohongmei@nchu.edu.cn (H.M.)

**Keywords:** hypergraph node classification, semi-supervised learning, spectral inductive bias, scale-aware modeling, adaptive fusion

## Abstract

Existing methods for hypergraph node classification usually rely on local message passing and use a unified strategy for topological modeling across hyperedges of different sizes. However, they have two limitations in semi-supervised settings. First, representation learning mainly depends on local neighborhoods, making it difficult to incorporate global topological information. Second, a unified structural modeling strategy cannot effectively handle both small and large hyperedges. Small hyperedges require modeling fine-grained local relations, while large hyperedges need sparse group-level structure. To address these issues, we propose S2-HGNN, a scale-aware hypergraph node classification framework with spectral inductive bias for semi-supervised learning. S2-HGNN first injects global topological information into the input features using complementary hypergraph spectral operators. It then constructs different auxiliary topologies based on hyperedge size. For small hyperedges, it uses Top-k constrained clique expansion to preserve representative local relations. For large hyperedges, it uses star expansion to reduce redundant connections while preserving sparse group-level structure. Finally, node representations are jointly learned from the original hypergraph backbone and the two auxiliary branches, and final predictions are obtained through node-level adaptive fusion. Experiments on multiple public datasets show that the proposed method consistently outperforms strong baselines and exhibits superior robustness under feature perturbations.

## 1. Introduction

Real-world relational data often exhibit intrinsic higher-order relations that cannot be adequately modeled by simple pairwise connections [1]. For example, a single paper in co-authorship networks [2] usually involves multiple authors. In co-citation networks [3], a set of documents may exhibit higher-order co-occurrence patterns due to citations, while in recommendation systems [4], users, items, and contextual factors often participate in multi-entity interactions. Compared with graphs, hypergraphs allow a single hyperedge to connect multiple nodes simultaneously, providing a more natural way to model such higher-order relations [5]. Hypergraph node classification has attracted increasing attention.

In recent years, hypergraph neural networks have advanced rapidly [6]. Yang et al. [7] systematically reviewed recent advances in hypergraph neural networks and highlighted that deep learning-based hypergraph representation learning has become a major research direction in this field. Feng et al. [8] were among the first to introduce neural networks into hypergraph learning by propagating information over hypergraph structures, laying the foundation for subsequent studies. Building on this line of research, Yadati et al. [9] proposed HyperGCN, which effectively reduces the computational cost of higher-order information propagation by approximating hyperedges with graph structures. Huang and Yang [10] provided a unified view of graph and hypergraph neural networks from the perspective of message passing and established a two-stage node–hyperedge–node propagation paradigm. Chien et al. [11] revisited hypergraph layer design from the perspective of set-function modeling, thereby enhancing the model’s representational capacity and generalization performance. Subsequently, researchers further advanced hypergraph representation learning through representation enhancement, feature encoding, and multi-view modeling. Wu et al. [12] introduced a collaborative contrastive learning mechanism to improve the consistency and discriminative power of hypergraph node representations. Zou et al. [13] proposed a general feature encoding framework that emphasizes the joint modeling of node attributes and topological structure. Saxena et al. [14] characterized hypergraph structures jointly from both spatial and spectral perspectives, thereby enhancing the model’s ability to capture complex higher-order relations. Zhang et al. [15] improved node classification performance in semi-supervised settings through multi-level information interaction and representation enhancement. Liang et al. [16] further pointed out that static and uniform topological assumptions do not always hold under heterophily or complex structural distributions. Existing studies have advanced hypergraph node classification from multiple perspectives, including propagation mechanism optimization, feature representation enhancement, and multi-view structural modeling. Consequently, local propagation over the original hypergraph structure and multi-view enhancement have gradually become representative research directions in this field. Different from heterophily-aware graph methods that mainly focus on label inconsistency among neighboring nodes, this work focuses on structural heterogeneity induced by hyperedge-size variations in hypergraphs. Nevertheless, both lines of research suggest that uniform propagation or topology construction may be insufficient under complex structural distributions.

Although existing methods have made significant progress in modeling higher-order relations, two key limitations remain in semi-supervised node classification. First, most methods rely heavily on local neighborhood propagation and fail to fully exploit global topological information. When labeled data are scarce, noisy local structures and incomplete node attributes can lead to unstable representations and weakened generalization performance [17]. Second, existing methods generally apply a uniform strategy for topological modeling and message propagation, without considering the structural heterogeneity caused by variations in hyperedge size. Small hyperedges are better suited to preserving fine-grained node relations, whereas large hyperedges are more likely to introduce redundant connections and amplify noise when they are treated with clique expansion [18]. A unified strategy risks either under-modeling fine-grained relations in small hyperedges or over-connecting unrelated nodes in large hyperedges.

Although spectral-domain methods [19], representation enhancement techniques [13], multi-view modeling [14], and topology expansion strategies have been explored, they are usually designed in isolation [20]. Spectral methods mainly emphasize global structural constraints, while topology expansion methods focus on local relation modeling [21]. As a result, the joint modeling of global topological information, scale-specific auxiliary structures and the higher-order semantics of the original hypergraph remains insufficiently explored.

A key observation behind this work is that the structural role of a hyperedge is closely related to its size. Existing spatial-spectral hypergraph methods typically perform spectral and spatial learning on a unified hypergraph structure, while existing topology expansion methods often apply the same expansion strategy to all hyperedges. However, such a unified design overlooks the different modeling requirements of small and large hyperedges. Small hyperedges are more suitable for preserving representative fine-grained local relations, whereas large hyperedges require sparse group-level modeling to avoid redundant dense connections. Therefore, instead of treating spectral modeling, topology expansion, and multi-branch learning as independent components, it is more desirable to integrate them under a scale-aware structural principle guided by hyperedge-size heterogeneity.

To address the above limitations, we propose S2-HGNN, a scale-aware hypergraph node classification framework with spectral inductive bias for semi-supervised learning. The proposed framework preserves higher-order semantics by using propagation over the original hypergraph as the backbone. Meanwhile, it constructs scale-specific auxiliary topologies according to hyperedge size: Top-k constrained clique expansion is applied to small hyperedges to capture representative fine-grained relations, while star expansion is applied to large hyperedges to reduce redundant connections and capture sparse group-level structures. In addition, S2-HGNN injects global topological information at the input stage through complementary hypergraph spectral operators, and fuses representations learned from different structural views through node-level adaptive fusion. Compared with general multi-view hypergraph models, the auxiliary views in S2-HGNN are not heuristically generated but are explicitly guided by the distinct structural roles of small and large hyperedges. The framework therefore jointly exploits global spectral information, original higher-order semantics, fine-grained local relations, and sparse group-level structures within a unified model. The main contributions are summarized as follows:

(1) We propose a scale-aware topological decomposition strategy. It constructs different auxiliary topologies based on hyperedge size, enabling modeling of fine-grained relations in small hyperedges and sparse group-level structure in large hyperedges. This improves the model’s ability to capture higher-order relations with scale heterogeneity.

(2) We design an input-level spectral inductive bias mechanism. By using complementary hypergraph spectral operators, it injects global topological information into the input features. This reduces the sensitivity of local propagation to noise and improves the exploitation of global structural information in semi-supervised settings, enhancing the stability and robustness of node representations.

(3) We construct a joint learning framework, called S2-HGNN, that integrates the original hypergraph backbone with multiple auxiliary branches. Through node-level adaptive fusion, it combines higher-order semantics, fine-grained local relations, sparse group-level structures and global topological information, improving classification performance under limited labeled data.

The remainder of this paper is organized as follows. Section 2 reviews the related work and presents the necessary preliminaries, including hypergraph node classification, spectral and spatial methods, and topological decomposition strategies. Section 3 introduces the proposed framework for hypergraph node classification with spectral inductive bias and scale-aware topological decomposition. Section 4 presents the experimental results and detailed analysis. Section 5 concludes the paper and outlines future research directions.

## 2. Preliminaries

### 2.1. Definitions of Hypergraphs

Let *G =* (*V*, *E*, *W*) denote an undirected hypergraph, where *V* = $\left\{v_{1},v_{2},\ldots ,v_{N}\right\}$ is the set of nodes and *E =* $\left\{e_{1},e_{2},\ldots ,e_{M}\right\}$ is the set of M hyperedges. Each hyperedge is a nonempty subset of V, that is, $e_{j}\neq \emptyset$. Let $H\in {\left\{0,1\right\}}^{N\times M\;}$ be the incidence matrix of the hypergraph, where $H_{ij}$ = 1 if $v_{i}\in e_{j}$ and $H_{ij}$ = 0 otherwise. $W=diag(w_{1},\ldots ,w_{M})$ is the hyperedge weight matrix [22]. In this paper, we focus on undirected and unweighted hypergraphs, and thus set W = I. For $v_{i}\in V$ and $e_{j}\in E$, the node degrees and hyperedge degrees are defined as $\begin{array}{c} d_{v_{i}}=\sum_{j=1}^{M}w_{j}H_{ij} \end{array}$ and $\delta_{e_{j}}=\sum_{i=1}^{N}H_{ij}$, respectively. $D_{v}\in R^{N\times N}$ and $D_{e}\in R^{M\times M}$ denote the diagonal matrices of node degrees and hyperedge degrees, respectively.

Node features are denoted by $X\in R^{N\times d}$, where d is the feature dimension. In the semi-supervised node classification task, let $\begin{array}{c} V_{train}\subseteq V \end{array}$ be the set of labeled nodes, and let $Y_{train}\in R^{|V_{train}|\times C}$ be the corresponding label matrix, where C is the number of classes. The objective is to learn node representations that jointly capture local structural semantics and global topological information under limited supervision, and to predict the class labels of unlabeled nodes.

### 2.2. Spatial and Spectral Methods for Hypergraph Neural Networks

Existing hypergraph node classification methods can generally be divided into two categories: spatial methods and spectral methods. The former rely on explicit message passing to learn node representations through information exchange between nodes and hyperedges, whereas the latter extract smoothed or convolutional features from the global topological structure by constructing hypergraph spectral operators. These two categories correspond to two different modeling paradigms: local higher-order propagation-based modeling and global structure-aware modeling. Together, they form the main technical foundation of current hypergraph representation learning [8].

#### 2.2.1. Spatial Hypergraph Neural Networks

Spatial methods typically follow a two-stage message-passing paradigm, namely node–hyperedge–node, in which information is first aggregated from nodes to hyperedges and then propagated back from hyperedges to nodes, thereby capturing higher-order topological relations. This modeling strategy is generally regarded as a natural extension of neighborhood aggregation and inductive representation learning in graph neural networks to the hypergraph setting [23]. A generic formulation can be written as $h_{e}={Aggr}_{v}\left(\left\{x_{i}|v_{i}\in e\right\}\right),\;\;{x_{i}}^{\prime }=Update\left(x_{i},{Aggr}_{e}\left(\left\{h_{e}|v_{i}\in e\right\}\right)\right)$, where $h_{e}\;$ denotes the aggregated representation of hyperedge e; ${Aggr}_{v}$ and ${Aggr}_{e}$ denote the aggregation functions from nodes to hyperedges and from hyperedges to nodes, respectively; and Update denotes the node representation update function.

These methods can directly leverage the original hypergraph structure for higher-order relationship propagation and show strong structural modeling ability [21]. Representative models under this paradigm include HNHN [24], HGNN+ [25], and the UniGNN framework [10], including UniSAGE and UniGAT. In particular, UniGNN [10] provides a unified formulation of the two-stage message-passing process, laying an important foundation for subsequent hypergraph neural network design. In addition, HyperSAGE [26] extends the idea of inductive representation learning to the hypergraph setting, thereby enhancing the model’s ability to generalize to unseen nodes and adapt to local neighborhood structures.

Spatial methods are effective at preserving local higher-order interactions in the original hypergraph. However, because their representation updates rely primarily on local propagation paths, their ability to exploit global topological patterns remains limited.

#### 2.2.2. Spectral Hypergraph Neural Networks

Spectral methods characterize the global topological structure of hypergraphs by constructing hypergraph spectral operators, which are then used to perform feature smoothing or spectral convolution [27]. Representative hypergraph spectral operators include the HGNN normalized propagation operator $\Theta_{hgnn}=D_{v}^{-1/2}H{WD}_{e}^{-1}H^{⊤}D_{v}^{-1/2}$ and the symmetric normalized Laplacian $\Delta_{sym}=I-\Theta_{hgnn}$. The random-walk propagation operator is defined as $P_{rw}=D_{v}^{-1}HWD_{e}^{-1}H^{⊤}$, and the corresponding random-walk Laplacian $\Delta_{rw}=I-P_{rw}$. These operators characterize the global structure of hypergraphs from different perspectives. $P_{rw}$ describes transition-probability-based diffusion, while $\Delta_{rw}$ represents the corresponding random-walk residual operator. Similarly, $\Theta_{hgnn}$ corresponds to symmetric normalized smoothing propagation, while $\Delta_{sym}$ represents the associated residual operator under symmetric normalization [28]. By incorporating such spectral operators, models can introduce global topological information at either the input stage or the message passing process, thereby addressing the limitations of local propagation in global modeling.

### 2.3. Hypergraph Topological Decomposition and Auxiliary Structure Modeling

The original hypergraph represents multi-way relations in a set-based form, which naturally preserves higher-order semantics, but does not directly expose pairwise relations among nodes [29]. To better capture local structural patterns, prior studies often transform hyperedges into graph structures for auxiliary modeling, among which clique expansion and star expansion are two representative strategies [30]. These two strategies have different modeling biases. Clique expansion preserves fine-grained pairwise relations, whereas star expansion compresses large-scale group structures. They are suitable for different scenarios in hypergraph node classification.

#### 2.3.1. Clique Expansion

Clique expansion decomposes each hyperedge into a complete subgraph by connecting every pair of nodes within the hyperedge, thereby preserving the fine-grained relational structure among its incident nodes. However, this strategy has a clear limitation: when hyperedges are large, clique expansion introduces many redundant connections, and its computational complexity grows quadratically with the hyperedge size, making it difficult to scale to hypergraphs with large hyperedges. To address this issue, previous studies have explored replacing full clique expansion with sparser graph approximations to reduce the computational overhead induced by large hyperedges.

#### 2.3.2. Star Expansion

Star expansion introduces a virtual center node for each hyperedge e and connects all nodes within the hyperedge to this virtual center, thereby forming a star-shaped topology. This strategy keeps the number of added edges linear in the hyperedge size, making it more suitable for scenarios involving large hyperedges. However, since information exchange mainly relies on the central node, star expansion often fails to preserve the fine-grained pairwise relations within a hyperedge.

Clique expansion and star expansion embody two distinct modeling biases. The former preserves fine-grained local relations but incurs higher computational cost, whereas the latter favors structural compression and efficient propagation but is less effective at preserving fine-grained relational details. For hypergraph data with diverse hyperedge sizes, a single expansion strategy often cannot strike a balance between structural expressiveness and computational efficiency.

## 3. Model

### 3.1. Problem Description

Based on the hypergraph formulation introduced in Section 2, we consider the semi-supervised hypergraph node classification task. Given an undirected hypergraph G=(V,E), a node feature matrix $X\in R^{N\times d}$, and a set of labeled nodes, the goal is to learn node representations under limited supervision and predict the labels of unlabeled nodes.

In semi-supervised hypergraph node classification, the model must not only exploit node attributes and local neighborhood information, but also capture the topological features induced by higher-order relations. The node–hyperedge–node propagation mechanism on the original hypergraph can effectively preserve higher-order interaction semantics. However, relying only on local propagation still leads to two challenges in semi-supervised settings. First, the model does not fully exploit global topological information, making it difficult to learn stable node representations when supervision is limited or local structures are perturbed by noise. Second, the structural relations associated with hyperedges of different sizes differ substantially, and a unified topological modeling scheme cannot simultaneously capture the fine-grained local relations in small hyperedges and the sparse group-level structure in large hyperedges.

To address these issues, we pursue the following modeling objectives: while preserving the higher-order semantics of the original hypergraph, we introduce global topological information and construct different auxiliary topologies for hyperedges of different sizes. By jointly modeling these complementary structural patterns, we aim to improve structural modeling ability and representation stability in semi-supervised node classification. The next section presents the proposed framework and the implementation details of each module.

### 3.2. Overall Framework

**Figure 1:**
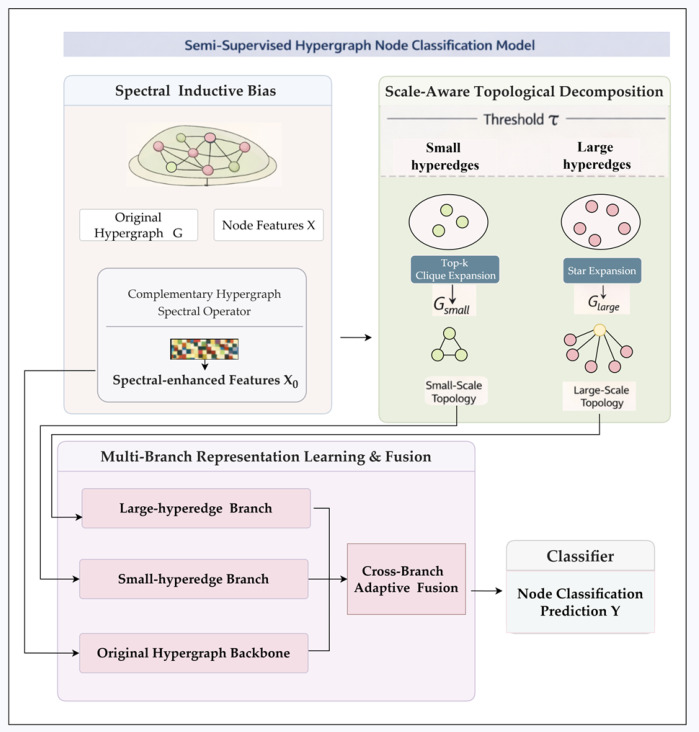
Illustration of the overall framework of the proposed method.

As illustrated in Figure 1, the proposed framework consists of four modules, namely, spectral inductive bias, scale-aware topological decomposition, multi-branch representation learning, and cross-branch fusion. It is designed to address two key challenges in semi-supervised hypergraph node classification: insufficient exploitation of global topological information and difficulty in modeling structural heterogeneity caused by differences in hyperedge size. Given node features and the original hypergraph, the model first enhances the input features with complementary spectral operators, thereby injecting global topological information into subsequent representation learning. It then constructs two types of auxiliary topologies based on hyperedge size, which are used to characterize the fine-grained local relations in small hyperedges and the sparse group-level structure in large hyperedges, respectively. Based on the original hypergraph and the two auxiliary views, the model jointly learns node representations through multiple branches. The final predictions are generated by node-level adaptive fusion across branches.

Rather than relying on a single local propagation mechanism, the proposed framework preserves the higher-order semantics of the original hypergraph. It models cross-scale structural relations through auxiliary topologies and introduces input-level spectral inductive bias to provide global topological information for different branches. This design enables the model to jointly exploit global spectral information, original higher-order semantics, and cross-scale structural information within a unified framework. The implementation details of each module are presented below.

### 3.3. Spectral Inductive Bias

Section 2.2 introduced the random-walk Laplacian, the symmetric normalized Laplacian, and the HGNN normalized propagation operator. Based on these spectral operators, we construct input-level spectral responses to inject global topological information before representation learning.

Given the node feature matrix X, we construct three spectral responses with complementary structural biases: ${\hat{X}}_{rw}=\Delta_{rw}X,\;{\hat{X}}_{sym}=\Delta_{sym}X,\;{\hat{X}}_{\mathrm{hg}}=\Theta_{\mathrm{hgnn}}X$. Here, ${\hat{X}}_{rw}$ captures random-walk-based residual information, ${\hat{X}}_{sym}$ captures high-frequency residual information under symmetric normalization, and ${\hat{X}}_{\mathrm{hg}}$ captures low-frequency smoothed information induced by the HGNN propagation operator.

It should be noted that $\Delta_{sym}\;=\;I-\Theta_{hgnn}$. Therefore, if the symmetric response were defined as $(I-\Delta_{sym})X$, it would be identical to $\Theta_{\mathrm{hgnn}}X$, leading to redundancy with the HGNN propagation response. To avoid this issue, we define ${\;\hat{X}}_{sym}=\Delta_{sym}X\;and\;{\hat{X}}_{\mathrm{hg}\;}={\;\Theta }_{\mathrm{hgnn}}X$. In this way,$\;{\;\hat{X}}_{sym}\;$ and ${\hat{X}}_{\mathrm{hg}}$ form a residual/smoothing decomposition under the symmetric normalized hypergraph operator, where ${\;\hat{X}}_{sym}$ captures the high-frequency residual component and ${\hat{X}}_{\mathrm{hg}}$ captures the low-frequency smoothed component. Together with ${\hat{X}}_{rw}$, these responses provide complementary structural biases for subsequent representation learning.

The three responses are then concatenated and projected into a unified spectral representation:

(1) $$X_{spec}=MLP\left(Concat({\hat{X}}_{rw},{\hat{X}}_{sym},{\hat{X}}_{\mathrm{hg}})\right)$$

where MLP(⋅) denotes a multi-layer perceptron and Concat(⋅) denotes feature concatenation. Although ${\hat{X}}_{sym}$ and ${\hat{X}}_{\mathrm{hg}}$ sum to X, concatenating them before the MLP keeps the high-frequency and low-frequency components separated, enabling the model to learn distinct nonlinear transformations for each. The final spectrally enhanced input representation is defined as:

(2) $$X_{0}=X+\lambda \cdot X_{spec}$$

where λ ∈ (0,1] is the spectral bias coefficient that controls the strength of global topological prior injection. The skip connection in $X_{0}=X+\lambda \cdot X_{spec}$ is used to preserve the original node attributes rather than to introduce another spectral response. Here, $X_{spec}$ acts as a topology-aware spectral correction, while X provides a stable feature baseline. The coefficient λ controls the contribution of the spectral correction.

Unlike simply stacking layers, the proposed spectral inductive bias globally enhances node features through spectral responses with complementary structural biases. The three responses, namely random-walk residual information, symmetric high-frequency residual information, and HGNN low-frequency smoothing information, provide diverse topological views that improve the model’s structural modeling capability and robustness in semi-supervised learning.

### 3.4. Scale-Aware Topological Decomposition

As discussed in Section 2.3, clique expansion and star expansion are suitable for different types of structural modeling. The former is more effective at preserving fine-grained local relations, but incurs relatively high computational complexity; the latter offers better scalability, but weakens direct pairwise interactions between nodes. Therefore, this paper does not adopt a single expansion strategy but instead constructs auxiliary topologies at specific scales based on the size of the hyperedges.

Let |e|  denote the size of hyperedge e. Given a threshold *τ*, the hyperedge set is partitioned into $E_{s}=\{e\in E\;|\;|e|\leq \tau \},\;E_{l}=\{e\in E\;|\;|e|>\tau \}$. Here, ${\;E}_{s\;}$ denotes the set of small hyperedges, and $E_{l}$ denotes the set of large hyperedges. The threshold τ is a key hyperparameter for controlling the scale partitioning, striking a balance between fine-grained local relationship modeling and sparse group-level structure learning. The smaller τ is, the more hyperedges are assigned to the large-hyperedge branch, reinforcing sparse group-level structure modeling; the larger τ is, the more hyperedges are assigned to the small-hyperedge branch, enhancing the modeling of fine-grained local relationships.

#### 3.4.1. Top-k Constrained Clique Expansion for Small Hyperedges

For small hyperedges, there are typically stronger local connections between nodes. If pairwise relations are ignored and the model relies on message passing over the original hypergraph, fine-grained structural information may be lost. However, performing full clique expansion over all node pairs would lead to quadratic computational complexity with respect to the hyperedge size. To address this issue, we adopt a Top-k constrained clique expansion in the small-hyperedge branch to retain the most representative local connections.

For each small hyperedge $e\in E_{s}$, we compute the similarity between any two nodes based on $X_{0}$. The similarity score is defined as follows:

(3) $$s_{ij}=\cos\left(x_{i}^{\left(0\right)},x_{j}^{\left(0\right)}\right)=\frac{x_{i}^{(0)⊤}\cdot x_{j}^{\left(0\right)}}{\left\|x_{i}^{\left(0\right)}\|\cdot \|x_{j}^{\left(0\right)}\right\|}$$

where $x_{i}^{\left(0\right)}\;and\;x_{j}^{\left(0\right)}$ denote the feature vectors of nodes $v_{i}\;and\;v_{j}$ in $X_{0}$ respectively. Then, for each node $v_{i\;}$ within hyperedge e, we retain only its Top-k most similar nodes, thereby constructing the Top-k constrained clique expansion edge set induced by hyperedge e. Let $A_{s}^{e}$ denote the Top-k clique expansion edge set generated by a small hyperedge e. To ensure symmetry, we treat the selected connections as undirected: if either $\left(v_{i},v_{j}\right)$ or $\left(v_{j},v_{i}\right)$ is selected, the undirected edge $\left(v_{i},v_{j}\right)$ is retained in the auxiliary topology. By merging the edge sets generated by all small hyperedges, we obtain the small-hyperedge auxiliary topology:

(4) $$G_{small}=\left(V,A_{s}\right),\;\;\;A_{s}=\underset{e\in E_{s}}{⋃}A_{s}^{e}$$

Compared with full clique expansion, this strategy does not seek to preserve all pairwise relations within each hyperedge. Instead, it prioritizes the preservation of more representative local connections, thereby achieving a better balance between structural modeling and computational complexity.

#### 3.4.2. Sparse Star Expansion for Large Hyperedges

For large hyperedges, using a dense expansion strategy would generate a large number of redundant edges and increase computational overhead. In the group relationships represented by large hyperedges, the core structure typically does not need to be expressed through pairwise node connections; instead, depicting group-level associations through virtual center nodes is often more efficient. This paper employs star expansion for sparse modeling on the large-hyperedge branch.

For any large hyperedge $e\in E_{l}$, we introduce a corresponding virtual center node $s_{e}$. Its initial representation is defined as follows:

(5) $$x_{s_{e}}=\frac{1}{\left|e\right|}\sum_{v_{i}\in e}x_{i}^{\left(0\right)}$$

where $x_{i}^{\left(0\right)}\;$ denotes the feature vector of node $v_{i}$ in the spectrally enhanced input representation $X_{0}$. Mean aggregation provides the virtual center node with a stable summary of the overall semantics of the hyperedge without introducing additional parameters.

We then connect all original nodes in hyperedge e to the virtual center node $s_{e}$, thereby forming a star-shaped subgraph. Let the set of all virtual center nodes be defined as $S=\{s_{e}|e\in E_{l}\}$. By merging the star-shaped subgraphs induced by all large hyperedges, the large-hyperedge auxiliary topology is formulated as follows:

(6) $$G_{large}=(V\cup S,A_{l})$$

where $A_{l}=\underset{e\in E_{l}}{⋃}\left\{(v_{i},s_{e})|v_{i}\in e\right\}$. Compared with clique expansion, this strategy does not restore all pairwise relations within large hyperedges. Instead, it achieves sparse group-level structures through virtual center nodes, effectively compressing redundant connections while preserving group-level association patterns. This star-shaped structure serves as an auxiliary topology to supplement large-hyperedge relationship modeling, while the original hypergraph backbone remains responsible for preserving the original higher-order semantics.

In summary, this paper constructs a scale-aware auxiliary topology complementary to the original hypergraph backbone by applying Top-k constrained clique expansion to small hyperedges and star-shaped expansion to large hyperedges. This design enhances the representation of fine-grained local relations and group-level structural patterns, respectively, providing a structural foundation better suited to the scale heterogeneity of hyperedges for subsequent multi-branch representation learning.

### 3.5. Joint Spatial-Spectral Representation Learning on Auxiliary Topology Branches

After constructing the small-hyperedge and large-hyperedge auxiliary topologies, we learn node representations on the two branches separately. Adjacency-based message passing is better suited for capturing local relationships, while spectral domain methods are more effective at capturing global information. This paper simultaneously learns spatial and spectral representations within each branch and achieves their adaptive coordination through a gating mechanism. The branch-level spectral learning is performed on the corresponding auxiliary topology, aiming to extract global structural information specific to each branch. In contrast, the input-level spectral inductive bias provides a shared global prior for all branches. These two approaches operate at different levels and are complementary.

Let m ∈ {*s,*l} denote the small-hyperedge branch or large-hyperedge topology branch, and let its adjacency matrix and degree matrix be denoted by $A_{m}\;and\;D_{m}$, respectively. The corresponding input features are denoted by $X_{m}^{\left(0\right)}$. Specifically, the input to the small-hyperedge branch is the spectrally enhanced representation of the original nodes, whereas the input to the large-hyperedge branch contains not only the original nodes but also the initial representations of the virtual center nodes.

#### 3.5.1. Spatial Representation Learning

We first extract local spatial information in each topology branch through adjacency-based message passing. Specifically, the spatial representation of branch m is computed as follows:

(7) $$H_{m}^{spa}=\sigma \left({\tilde{D}}_{m}^{-1/2}{\tilde{A}}_{m}{\tilde{D}}_{m}^{-1/2}X_{m}^{\left(0\right)}W_{m}^{spa}\right)$$

where ${\tilde{A}}_{m}=A_{m}+I$, ${\tilde{D}}_{m}$ is the corresponding degree matrix, and $W_{m}^{spa}$ is a learnable parameter matrix. This representation mainly captures the lower-order adjacency relations by topological expansion.

#### 3.5.2. Spectral Representation Learning

Relying solely on adjacency-based message passing makes it difficult to capture global structural information. To obtain the global structural features of each topological branch, we define its normalized Laplacian as follows:

(8) $$L_{m}=I-{\tilde{D}}_{m}^{-1/2}A_{m}{\tilde{D}}_{m}^{-1/2}$$

Based on this operator, we use a K-order polynomial filter [31] to extract spectral features. Specifically, the spectral representation of branch m is given by:

(9) $$H_{m}^{spec}=\sum_{r=0}^{K}\theta_{r}\cdot L_{m}^{r}X_{m}^{\left(0\right)}W_{m}^{fr}$$

where ${\;\theta }_{r}$ denotes the learnable filter coefficient, and $W_{m}^{fr}$ is the learnable projection parameter. This design approximates spectral filtering using sparse matrix multiplication, thereby avoiding the additional computational overhead associated with eigenvalue decomposition and enhancing the branch’s ability to capture global structural features.

#### 3.5.3. Intra-Branch Adaptive Fusion

Different nodes may rely on spatial and spectral representations to different extents. To account for this, we introduce a gating mechanism to perform adaptive fusion of the two types of representations. The gating weights are computed as follows:

(10) $$G_{m}=\sigma \left(W_{m}^{g}\left[H_{m}^{spa}∥H_{m}^{spec}∥X_{m}^{\left(0\right)}\right]+b_{m}^{g}\right)$$

where $W_{m}^{g}\;and\;b_{m}^{g}$ are the learnable parameters of the gating layer, $\left[\cdot ∥\cdot \right]$ denotes feature concatenation, and σ(⋅) denotes the sigmoid activation function.${\;G}_{m}\;$ has the same dimension as $H_{m}^{spa}\;and\;H_{m}^{spec}$, and is used for element-wise adaptive weighting of spatial and spectral representations.

Based on the gating weights, the fused branch representation is defined as follows:

(11) $$H_{m}=G_{m}⊙H_{m}^{spa}+(1-G_{m})⊙H_{m}^{spec}$$

where ⊙ denotes element-wise multiplication. For the large-hyperedge branch, since it contains virtual center nodes, we retain the representations of the original nodes corresponding to the first N rows, maintaining dimensional consistency with other branches. The auxiliary topological branches combine local adjacency information and global spectral structure into a complementary representation, offering feature representations for subsequent cross-branch fusion.

### 3.6. Higher-Order Semantic Representation Learning on the Original Hypergraph

Auxiliary topological branches are used to supplement local relationships and global information, while the higher-order semantics of the hypergraph are primarily preserved by the original hypergraph. Therefore, we retain the original hypergraph propagation backbone and perform standard node–hyperedge–node two-stage message passing on the original hypergraph, using the spectrally enhanced representation $X_{0}$ as the initial node features. Node representations are first aggregated into hyperedge representations, which are then propagated back to update node representations. For clarity, the two-stage propagation is written in its equivalent normalized matrix form. After $L_{h}$ propagation layers, the backbone representation on the original hypergraph is obtained as follows:

(12) $$\begin{array}{c} X_{h}^{\left(l+1\right)}=\sigma \left(D_{v}^{-1/2}HD_{e}^{-1}H^{⊤}D_{v}^{-1/2}X_{h}^{\left(l\right)}W_{h}^{\left(l\right)}\right), \end{array}\begin{array}{c} {\;\;\;\;\;\;\;H}_{h}=X_{h}^{\left(L_{h}\right)} \end{array}$$

where $X_{h}^{\left(l\right)}$ denotes the node representation at the *l-th* layer, $W_{h}^{\left(l\right)}$ is the learnable weight matrix, and σ(⋅) denotes the nonlinear activation function. This branch models the higher-order relations in the original hypergraph, providing a higher-order semantic representation for subsequent cross-branch fusion.

### 3.7. Cross-Branch Adaptive Fusion and Classification

Through the above modeling process, the model obtains three types of node representations: the original hypergraph backbone representation $H_{h}$, the small-hyperedge branch representation $H_{s}$, and the large-hyperedge branch representation $H_{l}$. These three representations correspond to higher-order semantics, fine-grained local relations, and sparse group-level structure.

To effectively integrate these, we design a node-level cross-branch fusion mechanism. For node $v_{i}$, let the three representations be denoted by $h_{i}^{h},\;h_{i}^{s}\;and\;h_{i}^{l}$, respectively. First, a learnable scoring function is employed to estimate the importance of each branch for the current node. The corresponding attention score is defined as follows:

(13) $$u_{i}^{\left(m\right)}=a_{m}^{⊤}\tanh\left(W_{f}^{\left(m\right)}h_{i}^{\left(m\right)}+b_{f}^{\left(m\right)}\right)\;m\in \{h,s,l\}$$

where $W_{f}^{\left(m\right)},b_{f}^{\left(m\right)}\;\mathrm{and}\;a_{m}$ are learnable parameters that map the representations from each branch into a unified attention score. A softmax normalization is applied over the branch dimension to obtain node-specific branch weights:

(14) $$\alpha_{i}^{\left(m\right)}=\frac{\exp\left(u_{i}^{\left(m\right)}\right)}{\sum_{n\in \{h,s,l\}}\exp\left(u_{i}^{\left(n\right)}\right)},\;\sum_{m\in \{h,s,l\}}\alpha_{i}^{\left(m\right)}=1$$

The final node representation is defined as:

(15) $$z_{i}=\sum_{m\in \{h,s,l\}}\alpha_{i}^{\left(m\right)}h_{i}^{\left(m\right)}$$

Stacking the representations of all nodes yields $Z={\left[z_{1},z_{2},\ldots ,z_{N}\right]}^{⊤}$. The final prediction is then produced through a linear classification head:

(16) $$\hat{Y}=softmax\left(ZW_{c}+b_{c}\right)$$

where $W_{c}$ and $b_{c}$ denote the classifier parameters. The model is trained with the cross-entropy loss, which is computed only over the labeled node set $V_{train}$. The loss function is given by:

(17) $$L=-\sum_{v_{i}\in V_{train}}\sum_{c=1}^{C}Y_{ic}{\log\hat{Y}}_{ic}$$

This design enables the model to adaptively coordinate higher-order semantics, local information, and global information across different nodes, thereby avoiding representation biases caused by fixed branch weights.

From the perspective of computational cost, the input-layer spectral inductive bias primarily performs feature transformation based on the sparse associations of the original hypergraph, and its complexity is approximately linear with respect to the total number of node–hyperedge associations. During the auxiliary topological construction process, branches with small hyperedges employ Top-k-constrained clique expansion to reduce the computational cost of local construction; conversely, branches with large hyperedges adopt star-shaped expansion, introducing a linear number of additional connections to avoid the connection redundancy caused by clique expansion. Overall, the proposed method preserves key local relations and group-structure information while maintaining a balance between structural modeling and computational cost.

## 4. Experiments

### 4.1. Experimental Settings

#### 4.1.1. Datasets

To evaluate the effectiveness of the proposed method in semi-supervised hypergraph node classification, we conduct experiments on five public benchmark datasets, namely, Citeseer (CC), Cora (CC), Pubmed (CC), Cora (CA), and DBLP (CA). Here, CC denotes hypergraphs constructed from paper co-citation relations, whereas CA denotes hypergraphs constructed from author co-authorship relations. These datasets cover two typical higher-order relational scenarios, namely, co-citation networks and co-authorship networks, and thus enable the evaluation of model performance from different perspectives. All datasets follow the commonly used public benchmark settings adopted in prior studies on hypergraph node classification [9].

In terms of data scale and structural characteristics, Citeseer (CC), Cora (CC), and Pubmed (CC) belong to co-citation networks, whereas Cora (CA) and DBLP (CA) correspond to co-authorship networks. These datasets differ substantially in terms of the number of nodes, hyperedge size, feature dimension, and structural sparsity. In particular, Pubmed (CC) is suitable for assessing the robustness of the model under sparse higher-order structures, while Cora (CA) can be used to examine the modeling stability and structural adaptability of the model on relatively large-scale co-authorship hypergraphs. These five datasets provide a comprehensive benchmark for validating the effectiveness of the proposed method across different higher-order relational scenarios. The details of the datasets are summarized in Table 1.

#### 4.1.2. Baselines

To comprehensively evaluate the effectiveness of the proposed method, we select representative baselines from different methodological paradigms, including non-structural baselines, classical hypergraph neural network models, and recent hypergraph learning methods.

We first include MLP as a non-structural baseline. This method performs classification based on node attributes without modeling the hypergraph structure and is used to measure the performance gains brought by higher-order relational modeling. We select HGNN [8], HyperGCN [9], and HNHN [24] as classical hypergraph neural network models, which represent typical technical paradigms including spectral propagation, graph-approximation-based modeling, and normalized higher-order information propagation, respectively. UniGCN [10] and AllSet [11] are included as strong representative baselines. UniGCN implements hypergraph modeling within a message-passing framework, while AllSet models the hyperedge aggregation process from the perspective of set functions. Additionally, UniG-Encoder [13] and DPHGNN [14] are introduced as representative advanced methods. The former emphasizes the collaborative encoding of node features and topological information, while the latter performs hypergraph representation learning from both spatial and spectral perspectives.

These baselines cover the modeling approaches in current hypergraph node classification research and enable an evaluation of the proposed method from multiple perspectives, including non-structural baselines, classical higher-order propagation models, unified message-passing models, and recent strong baselines.

### 4.2. Experimental Details and Evaluation Metric

All compared methods are evaluated under the same experimental protocol. For each dataset, we use the same node features, hypergraph structure, train/validation/test split, and set of random seeds for all methods. Training nodes are used for model optimization, validation nodes are used for early stopping and hyperparameter selection, and test nodes are used only for final evaluation. No test-label information is used during training, validation, or hyperparameter tuning.

For baselines with publicly available implementations, we use their official implementations whenever possible to avoid potential implementation bias. These implementations are adapted to our unified data splits and training pipeline without changing their core model architectures. For baselines without directly compatible implementations, we reimplement them according to the descriptions in the original papers.

For common hyperparameters, including learning rate, weight decay, dropout rate, hidden dimension, maximum number of epochs, and early-stopping patience, we use the same settings whenever applicable, or search them within the same candidate space. For method-specific hyperparameters, we follow the recommended ranges reported in the original papers and select the best configuration based on validation accuracy. The complete hyperparameter configuration is summarized in Table 2. Therefore, all methods are compared under a comparable hyperparameter tuning budget.

To reduce the influence of random initialization and data-order randomness, each method is independently run 10 times with the same set of random seeds, and the mean accuracy and standard deviation are reported. In addition, we conduct paired statistical significance tests between S2-HGNN and the strongest baseline on each dataset to further assess whether the observed improvements are statistically significant.

For evaluation, we adopt accuracy as the primary metric. Accuracy directly reflects the overall classification correctness on the test set and is the most commonly used evaluation criterion for hypergraph node classification. Compared with other metrics, Accuracy is better suited to the overall performance comparison considered in this work, as it provides an intuitive measure of the predictive performance of different methods in the semi-supervised node classification setting. For each independent run, Accuracy is computed on the test set, and the mean and standard deviation over 10 runs are reported.

### 4.3. Experimental Results and Analysis

This section compares S2-HGNN with several representative baselines on five public hypergraph node classification datasets. The results are summarized in Table 3, where classification accuracy is reported as mean ± standard deviation, and the bold values denote the best performance on each dataset.

To further validate the reliability of the observed improvements, we conduct statistical significance tests. Specifically, we compare S2-HGNN with the strongest baseline on each dataset using the paired Wilcoxon signed-rank test, based on the results from the same 10 independent runs under identical experimental settings. A significance level of 0.05 is adopted, and statistically significant improvements are marked with † in Table 3.

Table 3 reports the node classification results of the proposed method and various baselines on five public datasets. As shown in Table 3, the proposed method achieves the highest mean accuracy on Citeseer (CC), Cora (CC), Pubmed (CC), Cora (CA), and DBLP (CA), demonstrating its strong effectiveness across different hypergraph node classification scenarios. Compared with non-structural baselines and classical higher-order propagation models such as MLP, HGNN, HyperGCN, and HNHN, the proposed method exhibits more consistent improvements on most datasets. This suggests that relying on node attributes or local propagation mechanisms is often insufficient to fully characterize the complex higher-order relations in hypergraphs, whereas combining global topological information with scale-aware topological decomposition is beneficial for improving the quality of node representations.

Compared with strong baselines such as UniGCN, AllSet, UniG-Encoder, and DPHGNN, the proposed method shows more pronounced improvements on Pubmed (CC) and Cora (CA), indicating that the spectral inductive bias and scale-aware topological decomposition can yield greater benefits in scenarios with sparse higher-order structures or strong structural heterogeneity induced by variations in hyperedge sizes. The performance gains on some datasets are relatively limited, indicating that the degree of improvement also depends on dataset-specific structural characteristics, hyperedge size distributions, and attribute quality. The results show that the proposed method more effectively integrates global topological information and auxiliary topological information while preserving the higher-order semantics of the original hypergraph, thereby yielding more robust classification performance.

Moreover, we perform statistical significance tests to assess the reliability of the performance gains, which is particularly important when some improvements are relatively small compared with the standard deviations. The results show that S2-HGNN achieves statistically significant improvements over the strongest baselines on 3 out of 5 datasets under the paired Wilcoxon signed-rank test. Specifically, the improvements on Cora (CC), Pubmed (CC), and Cora (CA) are significant at the 0.05 level. This provides statistical evidence that the observed gains on these datasets are robust and unlikely to be caused by random variations in data splits or model initialization. On Citeseer (CC) and DBLP (CA), although S2-HGNN still achieves the highest mean accuracy, the differences do not reach statistical significance. This may be partly due to the relatively small performance margins, the observed variability, and the limited number of experimental runs. Overall, these results indicate that S2-HGNN consistently yields competitive or significantly better performance across diverse hypergraph node classification scenarios.

### 4.4. Analysis of Ablation Experiment Results

To rigorously evaluate the contribution of each key component, we conduct ablation studies with four degraded variants of S2-HGNN, as reported in Table 4.

The w/o SIB variant removes the input-level spectral inductive bias and directly uses the original node features as input. The w/o Scale Decomposition variant removes the scale-aware topological decomposition strategy. In this variant, hyperedges are no longer divided into small and large groups; instead, all hyperedges are modeled using a unified Top-k-constrained clique expansion. The auxiliary topology branch is still preserved, but it operates on a single-scale topology. The w/o Large-hyperedge Branch removes the large-hyperedge star expansion branch, while retaining the original hypergraph backbone and the small-hyperedge Top-k clique branch. The w/o Small-hyperedge Branch removes the small-hyperedge Top-k clique expansion branch, while retaining the original hypergraph backbone and the large-hyperedge star branch.

**Figure 2:**
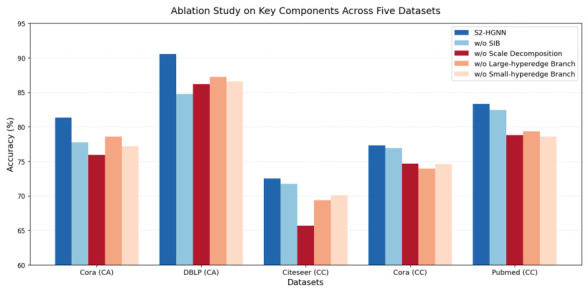
Ablation Study on Key Components Across Five Datasets.

As shown in Figure 2, S2-HGNN achieves the best performance on all five datasets, confirming that the proposed components jointly contribute to node classification. Removing the input-level spectral inductive bias causes an average accuracy drop of 2.28 percentage points, with larger drops on Cora(CA) and DBLP(CA). This indicates that injecting global topological information into the input features is beneficial, especially for datasets with more complex structures.

The w/o Scale Decomposition variant yields an average accuracy drop of 4.75 percentage points. This indicates that using a unified topology construction strategy for all hyperedges is insufficient. In particular, when large hyperedges are modeled in the same way as small hyperedges, redundant or noisy pairwise connections may be introduced, which can weaken the quality of message passing. Therefore, the results support the necessity of scale-aware topological decomposition, where small and large hyperedges are modeled by different auxiliary structures.

The results of w/o Large-hyperedge Branch and w/o Small-hyperedge Branch further demonstrate that the two auxiliary branches provide complementary information. Removing either branch consistently reduces performance compared with the full model, indicating that both the small-hyperedge Top-k clique branch and the large-hyperedge star branch are useful. Moreover, the relative importance of the two branches varies across datasets. Removing the large-hyperedge branch leads to larger drops on Citeseer(CC) and Cora(CC), whereas removing the small-hyperedge branch causes larger drops on Pubmed(CC), Cora(CA), and DBLP(CA). This suggests that different datasets benefit from different structural views. Overall, the complete S2-HGNN outperforms both single-branch variants on all datasets, verifying that the two branches are not redundant but mutually complementary.

### 4.5. Parameter Analysis

**Figure 3:**
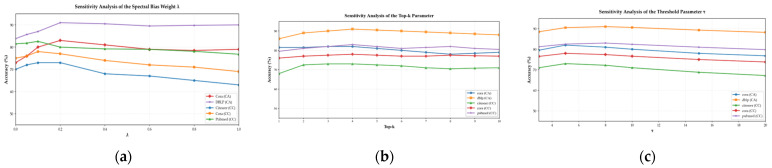
Sensitivity analysis of key hyperparameters on node classification performance (Accuracy): (**a**) the effect of the spectral bias weight λ; (**b**) the effect of the Top-k parameter; and (**c**) the effect of the threshold parameter τ.

To verify the reasonableness of the key parameter settings, we analyze the effects of the spectral bias weight λ, the Top-k parameter for local graph construction over small hyperedges, and the scale partition threshold τ on model performance. The experimental results are shown in Figure 3. In each experiment, one parameter is varied while all other parameters are fixed at their default values. It should be noted that these default settings are not optimal for every dataset; they are chosen by balancing cross-dataset performance and model stability.

For the spectral bias weight λ, the model generally exhibits a trend of first improving and then deteriorating on most datasets. This indicates that an appropriate amount of global topological information can enhance the spectral structural priors encoded in node representations and thereby improve classification performance. However, when λ becomes excessively large, overly strong spectral bias may weaken the contribution of the original attribute information and induce a certain degree of over-smoothing. Considering the results across all datasets, we finally set λ = 0.2 as the default value.

For the Top-k parameter k, different datasets exhibit some variation, but the overall trend reflects a trade-off between local connectivity sparsity and noise introduction. When k is too small, the locally clique-expanded subgraph becomes overly sparse, making it difficult to adequately model local relations within small hyperedges. As k increases, the model performance generally improves. However, when k becomes excessively large, more low-similarity connections are introduced, which may inject noise and weaken the generalization ability of the model. Considering both stability and overall performance, k = 4 performs robustly on most datasets and is therefore adopted as the default setting.

For the scale partition threshold τ, it mainly determines whether a hyperedge is assigned to the small-hyperedge branch or the large-hyperedge branch. When τ is too small, more hyperedges are assigned to the large-hyperedge branch, making it difficult to fully capture fine-grained local relations. In contrast, when τ is too large, too many hyperedges are assigned to the small-hyperedge branch, which weakens the functional distinction between branches at different sizes. The experimental results show that a moderate τ is more beneficial to model performance. Although the optimal values vary across datasets, the model is generally more stable in the middle range, and most datasets achieve their best or near-best results around τ = 5 or τ = 8. Considering both stability and overall performance, we finally adopt τ = 5 as the default value.

The model maintains relatively stable performance within a reasonable parameter range, and the proposed method does not rely excessively on delicate parameter tuning. The parameter analysis further demonstrates that the proposed method exhibits favorable parameter stability within a certain range, which is largely consistent with the trends observed in the overall performance and ablation studies above.

### 4.6. Feature-Noise Robustness Analysis

To evaluate the robustness of the proposed model under perturbed input features, we conduct feature-noise robustness experiments on three representative datasets, namely, Pubmed (CC), Cora (CA), and DBLP (CA). These datasets are selected because they cover the higher-order relational scenarios of co-citation and co-authorship networks, while also differing in node number, structural complexity, and hyperedge distribution characteristics. They provide a relatively comprehensive benchmark for assessing the model’s adaptability to feature perturbations under diverse structural conditions. We compare the proposed method with MLP, HGNN, and DPHGNN. Here, MLP reflects the degradation trend when relying on node attributes, HGNN serves as a classical hypergraph higher-order propagation model, and DPHGNN represents a recent dual-perspective hypergraph learning approach. This comparison allows us to assess the robustness of the proposed method to feature perturbations relative to representative baselines from different methodological categories.

**Figure 4:**
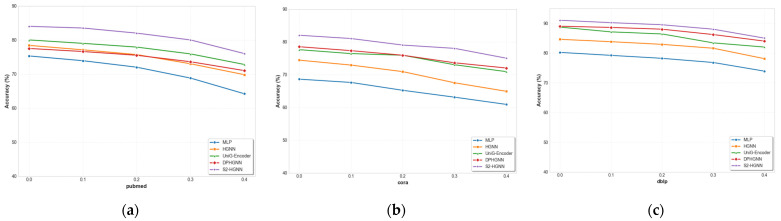
Feature perturbation robustness of the proposed method and baseline models under different perturbation ratios on three benchmark datasets: (**a**) Pubmed; (**b**) Cora; and (**c**) DBLP.

We apply random masking perturbations to the input feature matrix. For each node feature vector, a fixed proportion of feature dimensions is randomly selected and set to zero, simulating scenarios with missing or noise-corrupted attribute information. To systematically investigate the effect of different perturbation intensities, the masking ratio is set to 10%, 20%, 30%, and 40%. All other training settings are kept the same as described in Section 4.2, and the average classification accuracy is reported over multiple runs under each perturbation level. The experimental results are shown in Figure 4.

As the perturbation ratio increases, the classification performance of all methods declines to varying degrees, indicating that input feature noise has a generally adverse effect on hypergraph node classification. In contrast, the proposed method exhibits a consistently milder performance degradation, which is particularly evident on Pubmed (CC), Cora (CA), and DBLP (CA). Even when the original attribute information is corrupted, the proposed method can still maintain relatively stable discriminative performance.

This phenomenon can be attributed to two main factors. First, the input-level spectral inductive bias module introduces global topological information in addition to the original node features, enabling the model to acquire enhanced global topological awareness at an early stage of training and thereby reducing excessive reliance on local attributes. Second, scale-aware topological decomposition and multi-branch joint modeling provide complementary information from multiple structural views: the small-hyperedge branch helps preserve more discriminative fine-grained local relations, whereas the large-hyperedge branch offers a more stable sparse characterization of large-scale group structures. When features are perturbed, such joint modeling helps alleviate the performance degradation caused by the impairment of any single information channel.

A comparison of the performance degradation trends across methods on the three datasets shows that MLP is the most sensitive to feature perturbations, and relying solely on node attributes makes it difficult to maintain stable classification performance in noisy environments. Although HGNN can exploit hypergraph structural information, it mainly depends on a unified local higher-order propagation mechanism and thus remains vulnerable to unstable local patterns under feature perturbations. DPHGNN exhibits relatively strong robustness but still underperforms the proposed method overall. These results demonstrate that the synergy between spectral inductive bias and scale-aware topological decomposition can effectively improve the model’s adaptability to feature noise.

The feature-noise robustness experiments further demonstrate that the proposed method can maintain stable node classification performance even when the input features are perturbed, which is consistent with the trends observed in the overall performance and ablation studies.

### 4.7. Time Complexity Analysis and Training Time Analysis

To investigate the computational efficiency of S2-HGNN in hypergraph node classification, we conduct theoretical time complexity analysis and comparative experiments on training time with baseline methods. Table 5 and Table 6 report the theoretical complexity and training time comparison, respectively.

As shown in Table 5, the main computational cost of S2-HGNN comes from the original hypergraph backbone and the two auxiliary topology branches. The Top-k constrained clique expansion limits the number of retained local connections in small hyperedges, while the star expansion models large hyperedges with linear complexity. Compared with full clique expansion, whose complexity grows quadratically with the hyperedge size, the proposed scale-aware decomposition avoids dense pairwise construction on large hyperedges. Therefore, it controls the propagation cost while retaining representative local and group-level structural information. Since the auxiliary topologies are constructed before training, their construction costs are not included in the per-epoch training complexity.

## 5. Conclusions

This paper proposes S2-HGNN, a scale-aware hypergraph node classification framework with spectral inductive bias for semi-supervised learning. This framework introduces global topological information at the input stage and constructs different auxiliary topologies based on hyperedge size. It jointly models higher-order hypergraph semantics, fine-grained local relations within small hyperedges, and sparse group-level structures in large hyperedges. Experiments on multiple public datasets show that S2-HGNN achieves strong classification performance and remains robust under feature perturbations.

Despite its promising performance on multiple public datasets, the proposed framework still has several limitations. First, the scale partition threshold τ and the Top-k parameter k currently require tuning based on the validation set, and their optimal settings may vary across datasets, which increases the tuning cost. Second, the proposed scale-aware topological decomposition assumes noticeable heterogeneity in hyperedge sizes. When hyperedge sizes are relatively concentrated or structural differences between scales are weak, the benefits of this design may be limited. In addition, the present study mainly evaluates the proposed method on static hypergraph node classification tasks. Its applicability to large-scale and dynamic real-world hypergraph scenarios still requires further investigation. Future work will explore learnable scale partition mechanisms, more efficient auxiliary topology construction methods, and the generalization of the model to more complex hypergraph settings.

## Figures and Tables

**Table 1:** Statistics of the datasets. N represents the total number of nodes in the network, and E represents the total number of hyperedges. d indicates the dimension of features, and C denotes the number of classes.

Dataset	N	E	d	C
Citeseer (CC)	3312	1079	3703	6
Cora (CC)	2708	1579	1433	7
Pubmed (CC)	19,717	7963	500	3
Cora (CA)	2708	1072	1433	7
DBLP (CA)	41,302	22,363	1425	6

**Table 2:** Complete hyperparameter configuration.

Hyperparameter	Value	Description
$L_{h}$	2	Number of network layers
$d_{h}$	8	Dimension of hidden representations
*w_d_*	5 × 10^−4^	L2 regularization coefficient
*η*	0.01	Initial learning rate
*P_h_*	0.5	Dropout rate for hidden layers
*P_in_*	0.5	Dropout rate applied to the input layer
*P_att_*	0.6	Dropout rate applied to the attention mechanism
*E_m_*	400	Maximum number of training epochs
*P_es_*	200	Number of epochs without improvement before early stopping
*λ*	0.2	Weight of the spectral inductive bias term
*k*	4	Number of retained neighbors in local clique construction
*τ*	5	Threshold for hyperedge size partition
*N_r_*	10	Number of independent runs with different random seeds

**Table 3:** Evaluation results for node classification: mean accuracy (%) ± standard deviation. The best result on each dataset is shown in bold, and the strongest baseline is underlined. † indicates that S2-HGNN significantly outperforms the strongest baseline under the paired Wilcoxon signed-rank test (*p* < 0.05).

Methods	Citeseer (CC)	Cora (CC)	Pubmed (CC)	Cora (CA)	DBLP (CA)
MLP	64.09 ± 1.26	60.26 ± 1.45	75.27 ± 1.15	65.58 ± 1.54	80.24 ± 0.27
HGNN	69.72 ± 3.05	72.17 ± 2.86	78.36 ± 1.24	74.36 ± 2.81	84.62 ± 0.47
HyperGCN	59.66 ± 3.57	62.38 ± 3.27	74.72 ± 3.19	65.17 ± 1.29	82.58 ± 2.99
HNHN	68.23 ± 1.56	66.25 ± 1.06	76.22 ± 2.21	69.28 ± 2.40	83.27 ± 1.62
UniGCN	66.61 ± 1.12	73.99 ± 1.61	75.32 ± 1.12	74.34 ± 1.26	88.10 ± 0.85
AllSet	68.85 ± 1.56	75.08 ± 1.04	78.24 ± 0.83	76.25 ± 1.24	87.68 ± 1.69
UniG-Encoder	70.87 ± 1.13	74.57 ± 1.13	79.97 ± 1.19	77.56 ± 1.05	88.69 ± 2.85
DPHGNN	68.45 ± 3.28	69.68 ± 1.32	77.51 ± 0.84	78.48 ± 1.74	88.99 ± 1.80
S2-HGNN	**72.49** ± 1.14	**77.34** ± 1.97 †	**83.32** ± 1.75 †	**81.34** ± 1.49 †	**90.54** ± 0.72

**Table 4:** Ablation Results on Key Components.

Methods	Citeseer (CC)	Cora (CC)	Pubmed (CC)	Cora (CA)	DBLP (CA)
S2-HGNN	72.49 ± 1.14	77.34 ± 1.97	83.32 ± 1.75	81.34 ± 1.49	90.54 ± 0.72
w/o SIB	71.72 ± 1.33	76.94 ± 1.23	82.45 ± 1.03	77.76 ± 1.26	84.78 ± 0.82
w/o Scale Decomposition	65.66 ± 1.31	74.65 ± 1.82	78.81 ± 1.81	75.95 ± 1.14	86.19 ± 0.62
w/o Large-hyperedge Branch	69.35 ± 1.48	73.95 ± 1.76	79.38 ± 1.45	78.61 ± 1.57	87.25 ± 0.91
w/o Small-hyperedge Branch	70.08 ± 1.27	74.62 ± 1.64	78.56 ± 1.68	77.21 ± 1.79	86.58 ± 1.08

**Table 5:** Theoretical time complexity. N is the number of nodes, $d_{h}$ is the hidden dimension, $I=\sum_{e\in E}|e|$ is the total number of node–hyperedge incidences, $I_{s}$ and $I_{l}$ denote the total incidence sizes of small-hyperedge and large-hyperedge sets, respectively. K is the order of spectral filtering, k is the Top-k parameter, τ is the hyperedge size threshold, and $L_{h}$ is the number of hypergraph propagation layers.

Component	Time Complexity
Spectral Inductive Bias	$O(Id_{h}+Nd_{h}^{2})$
Small-hyperedge Topology Construction	$O(\tau I_{s}d_{h})$
Large-hyperedge Topology Construction	$O(I_{l}d_{h})$
Hypergraph Backbone Forward Pass	$O(L_{h}Id_{h})$
Small-hyperedge Auxiliary Branch	$O((K+1)kI_{s}d_{h})$
Large-hyperedge Auxiliary Branch	$O((K+1)I_{l}d_{h})$
Cross-branch Fusion	$O(Nd_{h})$
Total Training Per Epoch	$O(L_{h}Id_{h}+(K+1)kI_{s}d_{h}+(K+1)I_{l}d_{h}+Nd_{h}^{2})$

**Table 6:** Training time comparison (seconds). The results are reported as mean ± standard deviation over 5 independent runs. For the training time comparison, all methods are trained for 200 epochs under the same experimental setting. The training time includes model training and validation. The proposed method is highlighted in bold in the last row for clarity.

Methods	Citeseer (CC)	Cora (CC)	Pubmed (CC)	Cora (CA)	DBLP (CA)
MLP	0.62 ± 0.03	0.39 ± 0.02	0.48 ± 0.02	2.05 ± 0.08	6.32 ± 0.21
HGNN	1.08 ± 0.05	0.91 ± 0.04	1.26 ± 0.05	4.86 ± 0.18	13.72 ± 0.52
HyperGCN	1.14 ± 0.06	1.00 ± 0.05	1.45 ± 0.07	5.45 ± 0.24	15.80 ± 0.70
HNHN	1.66 ± 0.08	1.42 ± 0.06	2.02 ± 0.08	8.18 ± 0.31	23.40 ± 0.91
UniGCN	1.02 ± 0.05	0.86 ± 0.04	1.19 ± 0.05	4.38 ± 0.16	12.45 ± 0.47
AllSet	2.82 ± 0.13	2.41 ± 0.12	3.78 ± 0.16	15.60 ± 0.72	48.20 ± 2.35
UniG-Encoder	1.90 ± 0.09	1.64 ± 0.08	2.48 ± 0.11	9.60 ± 0.41	27.80 ± 1.10
DPHGNN	4.92 ± 0.31	4.35 ± 0.27	7.85 ± 0.48	31.18 ± 2.10	88.60 ± 6.20
S2-HGNN	**1.95 ± 0.09**	**1.70 ± 0.08**	**2.65 ± 0.10**	**10.80 ± 0.45**	**29.60 ± 1.25**

## Data Availability

The data presented in this study are available upon request from the corresponding author.
